# Visual memory profile in 22q11.2 microdeletion syndrome: are there differences in performance and neurobiological substrates between tasks linked to ventral and dorsal visual brain structures? A cross-sectional and longitudinal study

**DOI:** 10.1186/s11689-016-9174-5

**Published:** 2016-11-10

**Authors:** Mathilde Bostelmann, Maude Schneider, Maria Carmela Padula, Johanna Maeder, Marie Schaer, Elisa Scariati, Martin Debbané, Bronwyn Glaser, Sarah Menghetti, Stephan Eliez

**Affiliations:** 1 Office Médico-Pédagogique Research Unit, Department of Psychiatry, University of Geneva School of Medicine, Geneva, Switzerland; 2 Center for Contextual Psychiatry, Department of Neuroscience, KU Leuven, Leuven, Belgium; 3 Adolescence Clinical Psychology Research Unit, Faculty of Psychology and Educational Sciences, University of Geneva, Geneva, Switzerland; 4 Stanford Cognitive and Systems Neuroscience Laboratory, Stanford University School of Medicine, California, USA; 5 Research Department of Clinical, Educational and Health Psychology, University College London, London, UK; 6 Department of Genetic Medicine and Development, University of Geneva, Geneva, Switzerland; 7 Institute of Psychology, University of Lausanne, Lausanne, Switzerland

**Keywords:** 22q11.2 deletion syndrome, Visual memory, Visual cognitive development, Dorsal stream vulnerability hypothesis

## Abstract

**Background:**

Children affected by the 22q11.2 deletion syndrome (22q11.2DS) have a specific neuropsychological profile with strengths and weaknesses in several cognitive domains. Specifically, previous evidence has shown that patients with 22q11.2DS have more difficulties memorizing faces and visual-object characteristics of stimuli. In contrast, they have better performance in visuo-spatial memory tasks. The first focus of this study was to replicate these results in a larger sample of patients affected with 22q11.2DS and using a range of memory tasks. Moreover, we analyzed if the deficits were related to brain morphology in the structures typically underlying these abilities (ventral and dorsal visual streams). Finally, since the longitudinal development of visual memory is not clearly characterized in 22q11.2DS, we investigated its evolution from childhood to adolescence.

**Methods:**

Seventy-one patients with 22q11.2DS and 49 control individuals aged between 9 and 16 years completed the Benton Visual Retention Test (BVRT) and specific subtests assessing visual memory from the Children’s Memory Scale (CMS). The BVRT was used to compute spatial and object memory errors. For the CMS, specific subtests were classified into ventral, dorsal, and mixed subtests. Longitudinal data were obtained from a subset of 26 patients and 22 control individuals.

**Results:**

Cross-sectional results showed that patients with 22q11.2DS were impaired in all visual memory measures, with stronger deficits in visual-object memory and memory of faces, compared to visuo-spatial memory. No correlations between morphological brain impairments and visual memory were found in patients with 22q11.2DS. Longitudinal findings revealed that participants with 22q11.2DS made more object memory errors than spatial memory errors at baseline. This difference was no longer significant at follow-up.

**Conclusions:**

Individuals with 22q11.2DS have impairments in visual memory abilities, with more pronounced difficulties in memorizing faces and visual-object characteristics. From childhood to adolescence, the visual cognitive profile of patients with 22q11.2DS seems globally stable even though some processes show an evolution with time. We hope that our results will help clinicians and caregivers to better understand the memory difficulties of young individuals with 22q11.2DS. This has a particular importance at school to facilitate recommendations concerning intervention strategies for these young patients.

## Background

Chromosome 22q11.2 deletion syndrome (22q11.2DS), also known as DiGeorge syndrome [1] or velocardiofacial syndrome [2], affects 1:2000 live births [3] and exceeds 1:1000 in referrals for prenatal diagnosis [4]. In most cases, the syndrome results from a hemizygous 3-megabase microdeletion on the long arm of chromosome 22 [5]. The physical phenotype of 22q11.2DS includes craniofacial and cardiovascular abnormalities, immunodeficiency, short stature, hypocalcaemia, and brain abnormalities [3]. The syndrome is also frequently associated with psychiatric manifestations, such as attention-deficit/hyperactivity disorder, anxiety, mood disorders, and schizophrenia spectrum disorders emerging in adolescence and early adulthood [6].

Most studies report that individuals with 22q11.2DS show an intellectual level that falls in the borderline range (full-scale intellectual quotient (FSIQ) between 70 and 84), although a high heterogeneity is found in several cognitive domains [7]. Patients affected by the syndrome have specific difficulties in processing visual information such as faces, scenes, or geometrical stimuli [8–10]. Several authors even described 22q11.2DS as being characterized by the non-verbal learning disability syndrome (NLD), although some aspects of the verbal domain remain also impaired [11, 12]. Anatomically, the visual information processing system is separated in two hierarchically organized and specialized networks within the brain: a “ventral stream” and a “dorsal stream” [13]. Although the two pathways interact [14], the ventral stream, also known as the “what” pathway, relays information to the ventral and inferior temporal cortex including the fusiform gyrus and subserves object identification and face recognition [15, 16]. In contrast, the dorsal stream, known as the “where” pathway, relays information to the posterior parietal lobe and subserves spatial processing and visual control of spatially directed action [17, 18]. Memory abilities are also mediated by these two neural systems [19–22]. Activation of the inferior temporal cortex and the fusiform gyrus is observed when individuals have to retrieve and encode object identity of visual stimuli (e.g., shapes, colors, textures) or faces, whereas the parietal cortex and the supramarginal gyrus are activated during the encoding and retrieval of spatial localizations, object orientations, and volumetric properties of objects [23–25].

Previous studies highlighted significant visual memory impairments in individuals with 22q11.2DS [26, 27], such as difficulties in memorizing faces, shapes, line orientation, or spatial localizations [28–31]. Interestingly, the nature of the stimuli seems to influence the magnitude of the memory impairments. Evidences suggested that individuals with 22q11.2DS are more impaired in memory tasks related to the ventral stream compared to the tasks related the dorsal stream. Indeed, patients with 22q11.2DS have greater difficulties in memorizing faces or objects, whereas their memory of spatial localization seems partially impaired or even preserved [32–34]. However, these former studies have only included a limited number of tasks assessing the two streams and studied participants with a mean age ranging from 10 to 12.6 years. Further examination of this dissociation is therefore warranted. In addition, to our knowledge, no prior study investigated the relationship between these types of visual memory impairments in 22q11.2DS and their potential links with ventral and dorsal brain structures. On the neuronal level, abnormalities in temporal and parietal areas have been found in patients affected by t﻿he 22q11.2DS [35–38]. These abnormalities have been proposed to sustain the visual impairments observed in the syndrome.

In a developmental perspective, it is currently unknown whether these memory functions have an atypical development in 22q11.2DS. It has been shown that the cognitive profile is not stable in 22q11.2DS, with different patterns of strengths and weaknesses observed over time [39, 40]. In particular, although a global cognitive (FSIQ) decline has been found in patients affected, verbal and performance IQ scores were seen to evolve differently from childhood to adulthood, with performance IQ being more stable than verbal IQ [41, 42]. Looking more deeply within the non-verbal domain, Duijff et al. [43] showed a significant decline on the block design test in children with 22q11.2DS between 7.5 and 9.5 years, although other performance IQ subtests did not show any evolution. However, it is currently unclear how dorsal and ventral memory functions evolve during childhood in patients affected by the syndrome.

In the present study, we examined the capacity of subjects to memorize faces and spatial localizations. We also analyzed errors linked to failure of processing spatial or object characteristics of visual stimuli when subjects had to memorize and copy geometrical designs. Two kinds of investigation were carried out. The first set of hypothesis was made on a large sample of children and adolescents affected by the 22q11.2DS and on typically developing individuals aged between 9 and 16 years old. The first aim was to replicate the dissociation between tasks typically linked to the ventral stream and to the dorsal streams. Then, in light with a previous study showing that intellectual disability might have an impact on the neuropsychological profile of individuals with 22q11.2DS [34], additional analyses were performed to investigate the links between visual memory capacities and IQ measures. Finally, we also explored whether visual memory deficits in participants with 22q11.2DS were related to cerebral morphological alterations in the ventral (inferior and ventral temporal cortex) and dorsal brain areas (superior parietal cortex). Specifically, we made the following hypotheses: (1) individuals with 22q11.2DS would have lower scores in all visual memory tasks compared to typically developing individuals; (2) a specific visual memory profile would emerge, with greater impairments in tasks assessing the ventral stream; (3) in the 22q11.2DS group, IQ measures would be linked to visuo-spatial memory but not to visual-object memory; (4) altered visual memory would be related to changes in cortical morphology of underlying brain regions in participants with 22q11.2DS. Finally, as no previous study investigated this aspect in 22q11.2DS, the second aim of this study was to explore the longitudinal evolution of visual memory capacities between 9 and 16 years old (5).

## Methods

### Participants

The cross-sectional sample consisted of 71 participants with 22q11.2DS aged from 9 to 16 years (Table 1) assessed between February 2003 and March 2014. The Geneva cohort is also constituted of younger and older participants, but the measures included in the present study were available for this age range only. The presence of a 22q11.2 microdeletion was confirmed in all participants using quantitative fluorescent polymerase chain reaction (QF-PCR). Details about IQ measured by the Wechsler Intelligence Scale for Children-III (WISC-III) [44] are listed in Table 2. No statistical differences were found between participants wearing glasses (55 %) and those who did not (45 %) on any of the memory tasks (all *p* > 0.05). At the time of testing, 15 (19.7 %) participants were receiving psychotropic medication: 11 were on methylphenidate, 3 on antipsychotics, and 1 on antiepileptic medication. No individual had a diagnosis of psychotic disorder at the time of testing but 19 (26.8 %) were diagnosed with ADHD, 9 (12.7 %) with a generalized anxiety disorder, 6 (8.5 %) with a social phobia, and 2 (2.8 %) with a major depressive disorder. In order to examine the longitudinal evolution of visual memory over time, we selected all the participants who had at least two time points including one during childhood (9 to 12 years) and one during adolescence (13 to 16 years), leading to a total of 26 patients (T1 and T2; Table 1). Interval time between baseline (T1) and follow-up (T2) was on average 3.5 years (SD = 0.7).

**Table 1:** Cross-sectional and longitudinal samples’ description of 22q11.2DS and TD participants

	22q11.2DS participants			TD participants		
Samples	*N*	Mean age (SD)	Gender ratio	*N*	Mean age (SD)	Gender ratio
Cross-sectional samples	71	11.9 (2.01)	40 women (56 %)	49	11.62 (2.03)	25 women (51 %)
Longitudinal samples	26	T1: 11.31 (1.11)	15 women (58 %)	22	T1: 10.75 (0.88)	13 women (60 %)
		T2: 14.85 (1.01)			T2: 14.53 (0.58)

**Table 2:** Descriptive statistics and group comparison statistical differences for global cognitive measures, BVRT errors, and CMS types of subtests

	22q11.2DS participants *N* = 71		TD participants *N* = 49		Statistical differences between groups	Effect size
	Mean (SD)	Median	Mean (SD)	Median		
Global cognitive measures						
FSIQ	71.38 (11.85)		105.41 (9.00)		*p* < 0.001	*η2* = 0.71
PIQ	70.85 (11.43)		102.43 (12.5)		*p* < 0.001	*η2* = 0.63
VIQ	78.09 (14.19)		106.81 (9.73)		*p* < 0.001	*η2* = 0.56
BVRT errors						
Total of memory errors	7.75 (4.26)	7.00	4.29 (2.14)	4.00	*p* < 0.001	*r* = −0.42
Object memory errors	4.27 (3.30)	3.00	2.02 (1.36)	2.00	*p* < 0.001	*r* = −0.36
Spatial memory errors	2.73 (1.98)	3.00	1.82 (1.39)	2.00	*p* = 0.011	*r* = −0.23
Total of copy errors	3.06 (2.48)	2.00	1.18 (1.42)	1.00	*p* < 0.001	*r* = −0.42
Object copy errors	1.55 (1.96)	1.00	0.45 (0.77)	0.00	*p* < 0.001	*r* = −0.38
Spatial copy errors	1.45 (1.56)	1.00	0.73 (1.42)	0.00	*p* = 0.004	*r* = −0.26
CMS types of subtests						
CMS ventral subtests	6.47 (2.63)		10.20 (2.52)		*p* < 0.001	*η2* = 0.34
CMS dorsal subtests	7.71 (2.59)		10.47 (2.50)		*p* < 0.001	*η2* = 0.22
CMS mixed subtests	7.54 (2.76)		10.17 (2.61)		*p* < 0.001	*η2* = 0.19

Effects size for the non-parametric statistical tests were calculated based on the Rosental’s equation [85]

*FSIQ* full-scale intellectual quotient, *PIQ* performance intellectual quotient, *VIQ* verbal intellectual quotient

The cross-sectional comparison group consisted of 49 typically developing individuals (TD) aged between 9 and 16 years. The TD group consisted of siblings of participants with 22q11.2DS and community controls. All participants were screened by an experienced psychiatrist to ensure that none of them had any past or present neurological or psychiatric disorder. TD participants with a FSIQ above 120 were excluded from the analyses in order to have a comparison sample representative of the general population. The TD and 22q11.2DS groups did not differ regarding age (*t*(118) = −0.77, *p* = 0.4) or gender distribution (*x* ^*2*^ = 0.33, df = 1, *p* = 0.57). The longitudinal control sample was constituted of 22 TD participants aged between 9 and 12 years at T1 and between 13 and 16 years at T2. Interval time between T1 and T2 was on average 3.7 years (SD = 0.7).

Within-group comparisons showed that FSIQ and Performance IQ (PIQ) did not differ between T1 and T2 (all *p* > 0.05). However, a significant decreased of verbal IQ (VIQ) was found in the 22q11.2DS group between the two evaluations (*t*(25) = 2.43, *p* = 0.023) (Table 4). All participants of the two groups included in longitudinal analyses did not differ from the cross-sectional samples in terms of gender distribution (all *p* > 0.05).

### Materials

#### Benton Visual Retention Test

Parts A and C of the Benton Visual Retention Test (BVRT) [45] were administered in this order. In part A, the memory part, participants had to memorize ten designs printed on cards exposed for 10 s and reproduce them immediately. In part C, the copy part, participants had to copy the same designs while they remained in view. Errors were classified into five major categories: distortions, rotations, misplacements, size errors, and others. It should be noted that participants could make more than one error for the same design. To test the hypothesis that there are differences in the processing of ventral and dorsal information, error types were grouped into two main categories: spatial errors (misplacements and rotations errors) resulting in failure of processing visuo-spatial information and object errors (distortions and size errors) resulting in failure of processing visual-object information. The other types of errors (for example omissions or perseverations) were not included in the analysis as they are not related with spatial or object characteristics processing. The BVRT has been previously used in several pediatric populations, including children with learning disabilities [46, 47].

#### Children’s Memory Scale

We administered and analyzed subtests of the 9 to 16 years’ version of the Children’s Memory Scale (CMS) [48]. In the *Faces* subtest, the participants were asked to pay attention and remember a set of 16 faces. These faces had then to be recognized in another set of 36 faces mixing targets and distractors. The dependent variables used were the number of faces correctly identified immediately after the presentation of the stimuli and after a 30-min delay. In the *Dot localization* subtest, the participants had to recall the location of dots on a grid. Each stimulus was presented for 2 s. The dependent variables used were the number of correct dot locations across four trials and the number of correct dot locations following a 30-min delay (one trial). In the *Family Pictures* subtest, the participants were shown four pictures displaying different family scenes (e.g., a picnic scene) for 10 s. Each scene contained four characters appearing in different locations (top right, top left, bottom right, or bottom left) and engaged in specific actions. The dependent variables were the number of correct characters, locations, and actions correctly recalled immediately and after a 30-min delay. Finally, in the *Images Location* subtest, the participants had to memorize locations of images (animals and vehicles) on a grid. Each stimulus was presented for 2 s. The dependent variables used were the number of correct images’ locations across 16 trials. No delayed recall was available in this subtest. To test our hypothesis about the presence of differences in the ventral or dorsal visual memory processing in 22q11.2DS, we grouped the variables into three main categories: The *CMS dorsal subtests* score was composed of the mean standard scores of the *Dot Localization*’s delayed and immediate recalls and the standard scores of the *Image Localization’s* subtests. The *CMS ventral subtests* score was composed of the mean standard scores of *Faces’* delayed and immediate recalls together. Finally, a *CMS mixed subtests* score (i.e., subtests combining ventral and dorsal processing) was computed by averaging standard scores of the *Family Picture’s* delayed and immediate recalls. All the tests were performed by Master’s level psychologists according to the procedure described in the tests manuals [44, 45]. The CMS has been also previously used in 22q11.2DS [32, 49] and in children with learning disabilities [50].

#### Neuroimaging data acquisition

T1-weighted images were used to investigate brain morphological changes associated with impairments in visual memory in 22q11.2DS. Scans were acquired at the Geneva Center for Biomedical Imaging (CIBM) using a 1.5T and a 3T scanner. Scan parameters of the 1.5T Philips Intera scanner were 124 coronal slices, voxel size 0.94 × 0.94 × 1.5 mm, TR = 35 ms, TE = 6 ms, and flip angle = 45° and for the 3T Siemens Trio scanner, 192 coronal slices, voxel size 0.86 × 0.86 × 1.1 mm, TR = 2500, TE = 3 ms, and flip angle = 8°. High cross-scanner consistency of cortical thickness values was already reported in our previous study [51]. Twelve participants with 22q11.2DS were excluded from the original group of 71 patients because of the absence (*N* = 5) or the bad quality (*N* = 7) of the T1-weighted acquisition. These 12 subjects were excluded from the neuroimaging analyses only.

### Data analyses

#### Visual memory performances

Data were first checked for normality using the Kolmogorov-Smirnov test to conform to the assumptions of parametric tests. For the CMS variables, mixed, multivariate, and repeated-measures analyses of variance were used, followed by post hoc paired sample *t* tests to examine significant differences between pairs of variables. As the BVRT measures clearly violated normality assumptions in both groups of participants, non-parametric Mann-Whitney *U* tests for between-groups analyses and Wilcoxon signed-rank tests for within-groups analyses were performed. Thus, for the BVRT measures, medians were provided in results Tables 2 and 4. Spearman’s correlations were executed in the original cross-sectional sample to identify possible associations between the visual memory variables and global cognitive measures. Multiple comparisons corrections were applied for all the results using the Benjamini-Hochberg procedure [52].

#### Neuroimaging processing

**Fig. 1:**
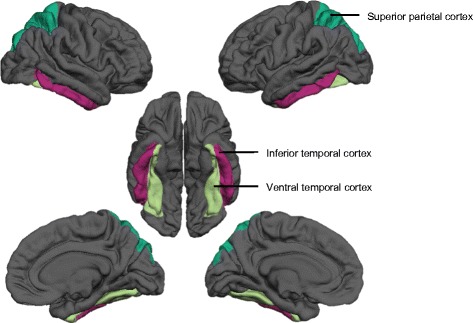
Regions of interest belonging to the dorsal (superior parietal cortex) and the ventral visual streams (inferior and ventral temporal cortex)

Neuroimaging data processing was conducted using the FreeSurfer software (http://surfer.nmr.mgh.harvard.edu). Three-dimensional cortical mesh models were reconstructed to obtain the interface between white and gray matter (white surface) and between gray matter and cephalospinal fluid (pial surface), and cortical thickness was measured as the distance between the two [53]. All surfaces were inspected and, if necessary, corrected by experienced users according to the gold-standard procedures. Cortical surfaces of each subject were resampled to an average spherical surface using an optimal registration algorithm based on the alignment of cortical folding patterns [54] and smoothed using a fill-width at half-maximum (FWHM) of 10 mm. Vertex-wise correlations were then computed between cortical thickness or local cortical volume and visual memory measures. Vertex-wise correlations were corrected for multiple comparisons using Monte Carlo permutation [55] at the threshold *p* < 0.05. In addition to these vertex-wise correlations, we also extracted cortical volume, total area, and average cortical thickness in specific regions of interest (ROI) corresponding to brain areas involved in the dorsal and ventral visual streams [13, 15]. For that purpose, we used the well-validated parcellation methodology by Desikan et al. [56] and selected regions of the superior parietal cortex as well as inferior and ventral (fusiform) temporal cortex (Fig. 1), which are involved in dorsal and ventral cognitive functions. Given the non-normal distribution of the BVRT error scores, we computed Spearman’s correlation coefficients between average cortical thickness, volume, and surface area in the superior parietal cortex and both BVRT spatial memory errors and CMS dorsal subtests scores. We further correlated the same morphometric measures in the inferior and ventral temporal cortex with the BVRT object memory errors and the CMS ventral subtests scores. Correlation analyses were conduced using age, gender, and scan type as covariates. As our aim was to associate the memory impairments observed in patients with 22q11DS with potential alterations in the ventral and dorsal streams, TD participants were not analyzed.

## Results

### Cross-sectional analyses

**Fig. 2:**
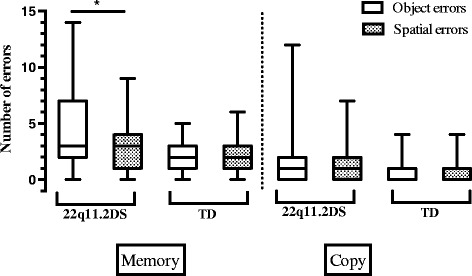
Boxplots with *whiskers* from minimum to maximum for object and spatial errors in the BVRT task of 22q11.2DS and TD groups (cross-sectional samples). The *top* of the box represents the 75th quartile, the *bottom* of the box represents the 25th quartile, and the *band* inside the box represents the median. * indicates significant values. Between-groups comparisons are not indicated on the graph, as all comparisons were statistically significant. Note: the medians for object and spatial copy errors for the TD group are equal to the 25th quartile

Descriptive statistics of the BVRT are shown in Table 2. Significant differences were found between 22q11.2DS and TD participants for all the measures. Within-groups analysis revealed that individuals with 22q11.2DS made significantly more object memory errors than spatial memory errors (*W* = −3.15, *p* = 0.002, *r* = −0.37) (Fig. 2). However, no significant difference was found between these two types of errors in the copy part. For TD participants, no significant differences were found between object and spatial errors in the memory part, or in the copy part. Overall, both groups made more memory than copy errors (all *p* < 0.001 and *r* > −0.80).

**Fig. 3:**
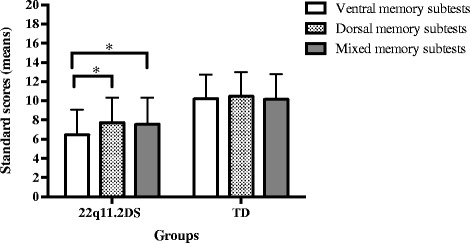
Standards scores with errors bars for the CMS subtests of 22q11.2DS and TD groups (cross-sectional samples). * indicates significant values. Between-groups comparisons are not marked in this graph, as all comparisons were statistically different

Descriptive statistics of the CMS variables are reported in Table 2. Between-groups differences in the CMS dorsal, ventral, and mixed subtests were investigated using a one-way multivariate analysis of variance (MANOVA). The multivariate test (*F*(3,116) = 32.34, *p* < 0.001, Wilks’ lambda = 0.55, partial *η2* = 0.45) and the individual independent variables were all significantly different between the two groups. Then, a one-way repeated measures analysis of variance (ANOVA) was conducted to compare the different types of CMS subtests within the 22q11.2DS group, revealing a significant effect of type of subtests (*F*(2,69) = 4.27, *p* = 0.011, Wilks’ lambda = 0.89, partial *η2* = 0.12). Separated *t* tests showed that individuals with 22q11.2DS had a better performance in CMS spatial subtests compared to CMS ventral subtests (*t*(70) = −2.87, *p* = 0.005, *η2* = −0.13) and a better performance in CMS mixed subtests compared to CMS ventral subtests (*t*(70) = −2.80, *p* = 0.007, *η2* = −0.13) (Fig. 3). No significant difference between the types of subtests was observed in the TD group.

Finally, correlation analyses between the visual memory variables and global cognitive measures were performed (Table 3). VIQ and PIQ were considered instead of the FSIQ because of the significantly lower PIQ compared to the VIQ in the 22q11.2DS groups (*t*(70) = −4.35, *p* < 0.001, *η2* = 0.21). For the 22q11.2DS group, correlations revealed that the number of BVRT object memory errors was negatively correlated to PIQ and VIQ. Also in the patient group, CMS ventral subtests and CMS mixed subtests were positively correlated to VIQ and PIQ, respectively.

**Table 3:** Spearman’s correlations coefficients between visual memory scores and global cognitive measures (PIQ and VIQ) for the 71 participants with 22q11.2DS and the 49 TD individuals

	BVRT		BVRT		CMS ventral subtests		CMS mixed subtests		CMS dorsal subtests	
	Object memory errors		Spatial memory errors							
	22q11DS	TD	22q11DS	TD	22q11DS	TD	22q11DS	TD	22q11DS	TD
PIQ	*−0.401*	−0.256	−0.234	−0.023	0.234	0.121	0.337	0.121	*0.352*	0.125
VIQ	*−0.375*	−0.079	−0.017	−0.401	0.252	0.262	*0.337*	0.167	0.315	0.183

Results in italics indicate statistical significance

*PIQ* performance intellectual quotient, *VIQ* verbal intellectual quotient

#### Correlations between ventral and dorsal memory and cortical morphology in participants with 22q11.2DS

After correction for multiple comparisons, BVRT object and spatial memory errors and CMS dorsal and ventral subtests scores did not show any significant correlation with values of cortical thickness and volume at the vertex-wise level. Similarly, at the ROI level, these measures were not significantly correlated with average cortical thickness, volume, and surface area in the superior parietal cortex and in the inferior and ventral temporal cortex (*N* = 59; all *p* > 0.05).

### Longitudinal analyses

**Fig. 4:**
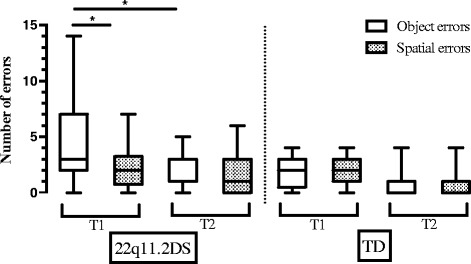
Boxplots with *whiskers* from minimum to maximum for object and spatial errors in the BVRT task of 22q11.2DS and TD groups (longitudinal samples). The *top* of the box represents the 75th quartile, the *bottom* of the box represents the 25th quartile, and the *band* inside the box represents the median. * indicates significant values. Between-groups comparisons are not marked in this graph. Note: when the median is not inside the boxplot, it is equal to the 25th quartile

The number of BVRT memory errors in both groups at T1 and T2 are reported in Table 4. Between-groups comparisons showed that participants with 22q11.2DS made more object memory errors than TD participants at T1 (*U* = 147.50, *z* = −2.99, *p* = 0.003, *r* = −0.43), and also at T2 (*U* = 172.50, *z* = −2.42, *p* = 0.016, *r* = −0.35). There were no between-group differences for spatial memory errors at T1 or at T2. Within-group comparison showed that participants with 22q11.2DS made more object memory errors at T1 compared to T2 (*W* = −2.19, *p* = 0.029, *r* = −0.43) (Fig. 4). There was no significant difference for spatial memory errors between the two visits. At T1, individuals with 22q11.2DS made more object memory errors than spatial memory errors (*W* = −2.31, *p* = 0.021, *r* = −0.45). At T2, the difference between object memory errors and spatial memory errors was no longer significant. For TD participants, no significant differences were found between T1 and T2 for object memory errors or spatial memory errors. Examination of the performance on the copy part showed that all participants made few errors. Both groups made also more errors on the memory parts than on the copy parts at T1 and T2, indicating that visual memory impairments were not solely due to visuo-constructive difficulties.

**Table 4:** Intellectual quotients scores, BVRT and CMS memory performances for 22q11.2DS and TD participants at T1 (9–12 years) and T2 (13–16 years)

	22q11.2DS participants (*N* = 26)					TD participants (*N* = 22)				
	T1		T2		Differences between T1 and T2	T1		T2		Differences between T1 and T2
	Mean (SD)	Median	Mean (SD)	Median		Mean (SD)	Median	Mean (SD)	Median	
Global cognitive measures										
FSIQ	74.65 (10.45)		73.00 (12.07)		ns	107.18 (9.13)		106.27 (9.78)		ns
PIQ	72.42 (12.86)		72.50 (11.52)		ns	103.00 (11.62)		103.82 (9.96)		ns
VIQ	82.54 (12.78)		78.92 (13.28)		*p* = 0.023	108.36 (11.14)		107.55 (9.45)		ns
BVRT errors										
Total memory errors	7.31 (4.21)	7.00	6.31 (4.11)	5.50	ns	4.14 (1.78)	4.00	2.77 (2.18)	2.50	ns
Object memory errors	4.38 (3.41)	3.00	2.85 (2.38)	2.50	*p* = 0.029	1.82 (1.30)	2.00	1.27 (0.99)	1.00	ns
Spatial memory errors	2.31 (1.85)	2.00	2.31 (2.18)	2.00	ns	1.86 (1.29)	2.00	1.32 (1.46)	1.00	ns
CMS types of subtests										
CMS ventral subtests	6.67 (3.04)		6.81 (2.78)		ns	10.41 (2.22)		11.09 (2.62)		ns
CMS dorsal subtests	7.53 (2.57)		7.37 (2.89)		ns	10.58 (2.44)		9.30 (2.56)		ns
CMS mixed subtests	7.37 (2.83)		7.75 (2.90)		ns	10.82 (2.51)		10.41 (2.77)		ns

*FSIQ *full-scale intelle﻿ctual quotient, *PIQ* performance intellectual quotient, *VIQ* verbal intellectual quotientns = non significant results

Descriptive statistics at T1 and T2 for the CMS subtests categories in both groups are also reported in Table 4. A three-way mixed ANOVA with diagnosis as between factor and types of subtests and time as within factors was performed. There was no significant interaction between the type of subtests and time. There was also no significant main effect of time. However, a principal effect of groups was found (*F*(1,49) = 56.0, *p* < 0.001, partial *η2* = 0.55). Group comparisons between 22q11.2DS and TD participants showed significant differences for all subtests at T1 and T2 (all *p* < 0.005, 0.19 < all partial *η2* < 0.33), except for the CMS dorsal subtests at T2. Finally, one-way repeated measures analyses of variance (ANOVA) were conducted and showed no significant differences between CMS subtests categories in both groups either at T1 or at T2.

## Discussion

In this study, we investigated visual memory in a large sample of 71 children and adolescents with 22q11.2DS and 49 typically developed participants. Specifically, we examined the performance on different memory tasks typically sustained by the visual ventral and the dorsal brain streams. We also made additional analysis in order to investigate links between memory performances and intellectual functioning. Furthermore, we explored whether the observed pattern of visual memory impairments in participants with 22q11.2DS was related to the morphology of specific brain regions. Finally, in a longitudinal perspective, we also investigated if visual memory abilities in 22q11.2DS evolved from childhood to adolescence.

### Copy impairments

Our results showed that individuals with 22q11.2DS were impaired when they have to copy geometrical figures. This result is in line with studies highlighting impairments in visuo-constructive abilities in children affected by the 22q11.2DS [10, 57]. We did not observe any dissociation between object and spatial errors, indicating no greater impairments in visual-spatial or visual-object processing when participants with 22q11.2DS had to copy. Visuo-constructive abilities depend on the integrity of multiple processes such as perception, visuo-spatial organization, motor functioning, and motivation [58, 59]. Consequently, many impaired processes also observed in 22q11.2DS could underly the observed difficulties during the copy part, such as spatial working memory deficits [60], executive impairments [61], perceptual [34, 62, 63], and psychomotor deficits [64]. Importantly, our results showed that both groups (22q11.2DS and TD) made more errors in the BVRT memory part compared to the copy part, indicating that the memory performance of individuals with 22q11.2DS is not only due to visuo-constructive impairments but also to memory contribution.

### Nature of memory impairments in 22q11.2DS

Overall, patients with 22q11.2DS were impaired in all memory tasks compared to the comparison group but were more impaired in memory tasks linked to the ventral stream compared to the tasks linked to the dorsal stream. Although Bearden et al. [49] reported an inverse pattern with greater impairments in visuo-spatial memory, these findings are generally consistent with the literature in 22q11.2DS [32–34]. Interestingly, visual ventral and dorsal functions have been previously investigated in other developmental disorders. As highlighted by Atkinson and Braddick [65], most of these studies showed a dorsal stream networks vulnerability rather than a ventral vulnerability [65–71], leading to the proposition that there might be a specific dorsal vulnerability across developmental disorders. However, considering the results of the present study, it appears that not all developmental conditions show this dissociation. Vicari et al. [72] also showed that patients with Down syndrome, another condition associated with intellectual disability but showing visual processing impairments, have better performance on tasks assessing visuo-spatial memory compared to visual-object memory. Thus, it is possible that in different neurodevelopment disorders from different genetic etiologies such as 22q11.2DS, Down syndrome, Williams syndrome, or Fragile X syndrome, the visual streams are affected differently throughout development. Further research collecting data using the same experimental design in individuals with various genetic disorders is clearly needed to better characterize the contribution of the two streams in the cognitive profile of genetic syndromes.

### Relationship between visual memory capacities and intellectual abilities in 22q11.2DS

A link between visual capacities and intellectual abilities was found in patients affected by the 22q11.2DS. In particular, visual-object memory was linked to IQ scores. In a recent study, Vicari et al. [34] found that short-term visuo-spatial memory but not visual-object memory was dependent of IQ. These conflicting results may be due to important differences between the tasks used in the two studies. In the present study, the tasks mainly relied on the encoding of visual aspects of stimuli, whereas Vicari et al. [34] used visuo-spatial and visual-object tasks both involving a serial number retrieval component, which is known to be impaired in 22q11.2DS [73]. We further observed that the encoding of visual-object stimuli was not only associated with PIQ but also with VIQ. This might suggest, as it was also suggested by Bearden et al. [49] and in accordance with our clinical observations, that patients with 22q11.2DS may use alternative verbal strategies to encode visual information (e.g., verbalizing “square” to remember a square). As our results also showed that children and adolescents with 22q11.2DS improved their performance in visual-object memory from childhood to adolescence, it is possible that they learned to use verbal strategies to encode visual information throughout development. This hypothesis should be examined in greater detail in future studies, as this may have important implications for implementing adequate remediation strategies for learning difficulties in patients with 22q11.2DS.

### Relationship between memory impairments and brain morphology

Contrary to our hypothesis, we did not observe any association between visual memory scores and cerebral alterations in the ventral and dorsal visual structures in patients with 22q11.2DS. This may suggest that the visual memory impairments observed in patients with 22q11.2DS are not related to brain morphological alterations, such as cortical volume, thickness, and surface area. However, other neuroimaging modalities, such as diffusion tensor imaging (DTI), may reveal the presence of a relationship between visual memory impairments and white matter connectivity in these patients. For example, Simon et al. [74] and Radoeva et al. [75] found that atypical structural connectivity in tracts belonging to the dorsal and ventral visual streams was correlated to visual processing impairments in patients with 22q11.2DS. Another possible reason explaining this lack of correlation is that the cognitive measures used in this study may not be specific enough, thus preventing to observe specific associations with areas of the ventral or dorsal stream [76]. In particular and regarding the link between IQ and visual memory, it is likely that these tasks involve other brain areas, such as the frontal lobe. This may be especially true if participants used encoding strategies during the memory tasks [77].

### Longitudinal development of cognitive profile in 22q11.2DS

From a developmental perspective, our results showed that whereas the capacities of patients with 22q11.2DS to memorize faces and spatial characteristics of visual stimuli were stable from childhood to adolescence, visual-object memory improved with time. This result can be interpreted in light of recent findings from the general population showing that the ventral and dorsal streams have different maturational rates. Indeed, the ventral areas keep developing until adulthood, whereas dorsal areas reach maturation earlier [78]. Behavioral studies also found some evidence that functions linked to the ventral and the dorsal streams mature at different rates [76, 79, 80]. Thus, one interpretation of the improvement of visual-object processing in 22q11.2DS is that, like in healthy children, ventral and dorsal functions show a different maturational rate, with a temporal delay of the ventral stream development. In this study, no longitudinal neuroimaging data were analyzed due to the unavailability of an adequate sample size. Therefore, a follow-up study would be needed to further explore this hypothesis.

### Limits and future directions

As mentioned earlier, the longitudinal analyses were performed in a relatively small sample of participants. However, we believe that using a longitudinal design limits the high inter-individual heterogeneity observed in patients with 22q11.2DS and therefore compensates for the small sample. One further limitation concerns the heterogeneity of the visual memory tasks used in the present study. Indeed, they involved different memory processes such as recollection or recognition that are potentially subserved by different brain networks. Besides, it would be interesting to investigate the influence of familiarity (“feeling of knowing”—a subjective experience of memory—versus “knowing”) on correct answers [81]. Thus, analyzing the specific contribution of different memory processes to the observed findings would be important. No significant difference between short- and long-term memory was found in this study. In the field of 22q11.2DS, there is currently no data available regarding very long-term memory consolidation (up to several days). Therefore, the presence of accelerated long-term forgetting (i.e., information that is encoded and retained normally over delays of up to 30-min but then forgotten at an abnormally rapid rate over longer periods) [82] may be under-detected in individuals with 22q11.2DS. Finally, a previous study has shown that psychotic symptoms, especially negative symptoms, are associated with the severity of visual memory deficits in 22q11.2DS [6]. As up to 80% of children and adolescents experience negative symptoms [83], it could be of great interest to further analyze their contribution to these results.

## Conclusions

In summary, we investigated visual memory in individuals affected by the 22q11.2DS and in typically developed individuals aged from 9 to 16 years. We identified that the magnitude of deficits in patients with 22q11.2DS was not identical depending on the nature of the stimuli, with increased difficulties in memorizing faces or shapes compared to visuo-spatial information. In contrast to our hypothesis, no significant anatomical associations with memory deficits were observed in patients with 22q11.2DS. Finally, we identified that the cognitive difficulties experienced by children with 22q11.2DS are not static, as object memory improved during the course of development. Knowing that 22q11.2DS have specific visual memory deficits with difficulties such as processing the shapes of the stimuli is important for selecting proper revalidation strategies and improving everyday lives of children suffering from genetic syndromes [84]. This has a particular impact for school materials that are essentially visual and that might be re-designed for patients with 22q11.2DS (for example simplifying visual information or promote verbal strategies in learning process).

## Abbreviations

22q11.2DS: 22q11.2 deletion syndrome
BVRT: Benton Visual Retention Test
CMS: Children’s Memory Scale
DTI: Diffusion tensor imaging
FSIQ: Full-scale intellectual quotient
IQ: Intelligence quotient
MRI: Magnetic resonance imaging
NLD: Non-verbal learning disability syndrome
PIQ: Performance intellectual quotient
QF-PCR: Quantitative fluorescent polymerase chain reaction
TD: Typically developed children
VIQ: Verbal intellectual quotient
WISC-III: Wechsler Intelligence Scale for Children, third version

## References

[1] Kirkpatrick JA, Digeorge AM (1968). Congenital absence of thymus. Am J Roentgenol Radium Ther.

[2] Shprintzen RJ, Goldberg RB, Lewin ML, Sidoti EJ, Berkman MD, Argamaso RV (1978). New syndrome involving cleft-palate, cardiac anomalies, typical facies, and learning-disabilities—velo-cardio-facial syndrome. Cleft Palate J.

[3] Shprintzen RJ (2008). Velo-cardio-facial syndrome: 30 years of study. Dev Disabil Res Rev.

[4] Grati FR, Gomes DM, Ferreira JCPB, Dupont C, Alesi V, Gouas L (2015). Prevalence of recurrent pathogenic microdeletions and microduplications in over 9500 pregnancies. Prenat Diagn.

[5] Carey AH, Kelly D, Halford S, Wadey R, Wilson D, Goodship J (1992). Molecular genetic study of the frequency of monosomy 22q11 in DiGeorge syndrome. Am J Hum Genet.

[6] Schneider M, Debbané M, Bassett AS, Chow EW, Fung WL, van den Bree M (2014). Psychiatric disorders from childhood to adulthood in 22q11.2 deletion syndrome: results from the International Consortium on Brain and Behavior in 22q11.2 Deletion Syndrome. Am J Psychiatry.

[7] De Smedt B, Devriendt K, Fryns JR, Vogels A, Gewillig M, Swillen A (2007). Intellectual abilities in a large sample of children with velo-cardio-facial syndrome: an update. J Intellect Disabil Res.

[8] McCabe K, Rich D, Loughland CM, Schall U, Campbell LE (2011). Visual scanpath abnormalities in 22q11.2 deletion syndrome: is this a face specific deficit?. Psychiatry Res.

[9] Glaser B, Debbané M, Ottet MC, Vuilleumier P, Zesiger P, Antonarakis SE (2010). Eye gaze during face processing in children and adolescents with 22q11.2 deletion syndrome. J Am Acad Child Adolesc Psychiatry.

[10] Antshel KM, Peebles J, AbdulSabur N, Higgins AM, Roizen N, Shprintzen R (2008). Associations between performance on the Rey-Osterrieth Complex Figure and regional brain volumes in children with and without velocardiofacial syndrome. Dev Neuropsychol.

[11] Swillen A, Vandeputte L, Cracco J, Maes B, Ghesquiere P, Devriendt K (1999). Neuropsychological, learning and psychosocial profile of primary school aged children with the velo-cardio-facial syndrome (22q11 deletion): evidence for a nonverbal learning disability?. Child Neuropsychol.

[12] Debbané M, Glaser B, Eliez S (2008). Encoding and retrieval processes in velo-cardio-facial syndrome (VCFS). Neuropsychology.

[13] Ungerleider LG, Haxby JV (1994). “What” and “where” in the human brain. Curr Opin Neurobiol.

[14] Van Essen DC, Anderson CH, Felleman DJ (1992). Information-processing in the primate visual-system—an integrated systems perspective. Science.

[15] Creem SH, Proffitt DR (2001). Defining the cortical visual systems: “what”, “where”, and “how”. Acta Psychol (Amst).

[16] Kravitz DJ, Saleem KS, Baker CI, Ungerleider LG, Mishkin M (2013). The ventral visual pathway: an expanded neural framework for the processing of object quality. Trends Cogn Sci.

[17] Rizzolatti G, Fogassi L, Gallese V (1997). Parietal cortex: from sight to action. Curr Opin Neurobiol.

[18] Zachariou V, Klatzky R, Behrmann M (2014). Ventral and dorsal visual stream contributions to the perception of object shape and object location. J Cogn Neurosci.

[19] Stern CE, Corkin S, Gonzalez RG, Guimaraes AR, Baker JR, Jennings PJ (1996). The hippocampal formation participates in novel picture encoding: evidence from functional magnetic resonance imaging. Proc Natl Acad Sci U S A.

[20] O’keefe J, Nadel L (1978). Hippocampus as a cognitive map. Psychol Med.

[21] Moscovitch M, Nadel L, Winocur G, Gilboa A, Rosenbaum RS (2006). The cognitive neuroscience of remote episodic, semantic and spatial memory. Curr Opin Neurobiol.

[22] Courtney SM, Ungerleider LG, Keil K, Haxby JV (1996). Object and spatial visual working memory activate separate neural systems in human cortex. Cereb Cortex.

[23] Moscovitch M, Rosenbaum RS, Gilboa A, Addis DR, Westmacott R, Grady C (2005). Functional neuroanatomy of remote episodic, semantic and spatial memory: a unified account based on multiple trace theory. J Anat.

[24] Moscovitch M, Kapur S, Kohler S, Houle S (1995). Distinct neural correlates of visual long-term-memory for spatial location and object identity—a positron emission tomography study in humans. Proc Natl Acad Sci U S A.

[25] Kravitz DJ, Saleem KS, Baker CI, Mishkin M (2011). A new neural framework for visuospatial processing. Nat Rev Neurosci.

[26] Niarchou M, Zammit S, van Goozen SH, Thapar A, Tierling HM, Owen MJ (2014). Psychopathology and cognition in children with 22q11.2 deletion syndrome. Br J Psychiatry.

[27] Wong LM, Riggins T, Harvey D, Cabaral M, Simon TJ (2014). Children with chromosome 22q11.2 deletion syndrome exhibit impaired spatial working memory. Am J Intellect Dev Disabil.

[28] Gur RE, Yi JJ, McDonald-McGinn DM, Tang SX, Calkins ME, Whinna D (2014). Neurocognitive development in 22q11.2 deletion syndrome: comparison with youth having developmental delay and medical comorbidities. Mol Psychiatry.

[29] Goldenberg PC, Calkins ME, Richard J, McDonald-McGinn D, Zackai E, Mitra N (2012). Computerized neurocognitive profile in young people with 22q11.2 deletion syndrome compared to youths with schizophrenia and at-risk for psychosis. Am J Med Genet B Neuropsychiatr Genet.

[30] Lepach AC, Petermann F (2011). Nonverbal and verbal learning: a comparative study of children and adolescents with 22q11 deletion syndrome, non-syndromal nonverbal learning disorder and memory disorder. Neurocase.

[31] Woodin M, Wang PP, Aleman D, McDonald-McGinn D, Zackai E, Moss E (2001). Neuropsychological profile of children and adolescents with the 22q11.2 microdeletion. Genet Med.

[32] Campbell LE, Azuma R, Ambery F, Stevens A, Smith A, Morris RG (2010). Executive functions and memory abilities in children with 22q11.2 deletion syndrome. Aust N Z J Psychiatry.

[33] Lajiness-O’Neill RR, Beaulieu I, Titus JB, Asamoah A, Bigler ED, Bawle EV (2005). Memory and learning in children with 22q11.2 deletion syndrome: evidence for ventral and dorsal stream disruption?. Child Neuropsychol.

[34] Vicari S, Mantovan M, Addona F, Costanzo F, Verucci L, Menghini D (2012). Neuropsychological profile of Italian children and adolescents with 22q11.2 deletion syndrome with and without intellectual disability. Behav Genet.

[35] Glaser B, Schaer M, Bemey S, Debbané M, Vuilleumier P, Eliez S (2007). Structural changes to the fusiform gyrus: a cerebral marker for social impairments in 22q11.2 deletion syndrome?. Schizophr Res.

[36] Andersson F, Glaser B, Spiridon M, Debbané M, Vuilleumier P, Eliez S (2008). Impaired activation of face processing networks revealed by functional magnetic resonance imaging in 22q11.2 deletion syndrome. Biol Psychiatry.

[37] Kikinis Z, Makris N, Finn CT, Bouix S, Lucia D, Coleman MJ (2013). Genetic contributions to changes of fiber tracts of ventral visual stream in 22q11.2 deletion syndrome. Brain Imaging Behav.

[38] Kikinis Z, Asami T, Bouix S, Finn CT, Ballinger T, Tworog-Dube E (2012). Reduced fractional anisotropy and axial diffusivity in white matter in 22q11.2 deletion syndrome: a pilot study. Schizophr Res.

[39] Maeder J, Schneider M, Bostelmann M, Debbané M, Glaser B, Menghetti S, et al. Developmental trajectories of executive functions in 22q11.2 deletion syndrome. J Neurodev Disord. 2016;8:10. 10.1186/s11689-016-9141-1 PMC4807556 27018204

[40] Gothelf D, Schneider M, Green T, Debbané M, Frisch A, Glaser B (2013). Risk factors and the evolution of psychosis in 22q11.2 deletion syndrome: a longitudinal 2-site study. J Am Acad Child Adolesc Psychiatry.

[41] Gothelf D, Eliez S, Thompson T, Hinard C, Penniman L, Feinstein C (2005). COMT genotype predicts longitudinal cognitive decline and psychosis in 22q11.2 deletion syndrome. Nat Neurosci.

[42] Vorstman JAS, Breetvelt EJ, Duijff SN, Eliez S, Schneider M, Jalbrzikowski M (2015). Cognitive decline preceding the onset of psychosis in patients with 22q11.2 deletion syndrome. Jama Psychiatry.

[43] Duijff SN, Klaassen PW, de Veye HF, Beemer FA, Sinnema G, Vorstman JA (2012). Cognitive development in children with 22q11.2 deletion syndrome. Br J Psychiatry.

[44] Wechsler D (1991). Wechsler Intelligence Scale for Children.

[45] Sivan AB (1992). Benton Visual Retention Test (5th Ed.).

[46] Snow JH (1998). Clinical use of the Benton Visual Retention Test for children and adolescents with learning disabilities. Arch Clin Neuropsychol.

[47] Rowley VN, Baer PE (1961). Visual-retention test-performance in emotionally-disturbed and brain-damaged children. Am J Orthopsychiatry.

[48] Cohen MJ (1997). Children’s memory scale.

[49] Bearden CE, Woodin MF, Wang PP, Moss E, McDonald-McGinn D, Zackai E (2001). The neurocognitive phenotype of the 22q11.2 deletion syndrome: selective deficit in visual-spatial memory. J Clin Exp Neuropsychol.

[50] Liddell GA, Rasmussen C (2005). Memory profile of children with nonverbal learning disability. Learn Disabil Res Pract.

[51] Mutlu AK, Schneider M, Debbané M, Badoud D, Eliez S, Schaer M (2013). Sex differences in thickness, and folding developments throughout the cortex. Neuroimage.

[52] Thissen D, Steinberg L, Kuang D (2002). Quick and easy implementation of the Benjamini-Hochberg procedure for controlling the false positive rate in multiple comparisons. J Educ Behav Stat.

[53] Fischl B, Dale AM (2000). Measuring the thickness of the human cerebral cortex from magnetic resonance images. Proc Natl Acad Sci U S A.

[54] Fischl B, Sereno MI, Dale AM (1999). Cortical surface-based analysis II: inflation, flattening, and a surface-based coordinate system. Neuroimage.

[55] Hagler DJ, Saygin AP, Sereno MI (2006). Smoothing and cluster thresholding for cortical surface-based group analysis of fMRI data. Neuroimage.

[56] Desikan RS, Segonne F, Fischl B, Quinn BT, Dickerson BC, Blacker D (2006). An automated labeling system for subdividing the human cerebral cortex on MRI scans into gyral based regions of interest. Neuroimage.

[57] Oskarsdottir S, Persson C, Eriksson BO, Fasth A (2005). Presenting phenotype in 100 children with the 22q11 deletion syndrome. Eur J Pediatr.

[58] Haggard P (2005). Conscious intention and motor cognition. Trends Cogn Sci.

[59] Loring DW, Martin RC, Meador KJ (1990). Psychometric construction of the Rey-Osterrieth complex figure: methodological considerations and interrater reliability. Arch Clin Neuropsychol.

[60] Sobin C, Kiley-Brabeck K, Daniels S, Khuri J, Taylor L, Blundell M (2005). Neuropsychological characteristics of children with the 22q11 deletion syndrome: a descriptive analysis. Child Neuropsychol.

[61] Shapiro HM, Tassone F, Choudhary NS, Simon TJ (2014). The development of cognitive control in children with chromosome 22q11.2 deletion syndrome. Front Psychol.

[62] Swillen A, Vogels A, Devriendt K, Fryns JP (2000). Chromosome 22q11 deletion syndrome: update and review of the clinical features, cognitive-behavioral spectrum, and psychiatric complications. Am J Med Genet.

[63] Henry JC, van Amelsvoort T, Morris RG, Owen MJ, Murphy DGM, Murphy KC (2002). An investigation of the neuropsychological profile in adults with velo-cardio-facial syndrome (VCFS). Neuropsychologia.

[64] Van Aken K, Caeyenberghs K, Smits-Engelsman B, Swillen A (2009). The motor profile of primary school-age children with a 22q11.2 deletion syndrome (22q11.2ds) and an age- and Iq-matched control group. Child Neuropsychol.

[65] Atkinson J, Braddick O (2011). From genes to brain development to phenotypic behavior: “dorsal-stream vulnerability” in relation to spatial cognition, attention, and planning of actions in Williams syndrome (WS) and other developmental disorders. Prog Brain Res.

[66] Braddick O, Atkinson J, Wattam-Bell J (2003). Normal and anomalous development of visual motion processing: motion coherence and ‘dorsal-stream vulnerability’. Neuropsychologia.

[67] Bellugi U, Lichtenberger L, Mills D, Galaburda A, Korenberg JR (1999). Bridging cognition, the brain and molecular genetics: evidence from Williams syndrome. Trends Neurosci.

[68] Atkinson J, King J, Braddick O, Nokes L, Anker S, Braddick F (1997). A specific deficit of dorsal stream function in Williams’ syndrome. Neuroreport.

[69] Kogan CS, Bertone A, Cornish K, Boutet I, Kaloustian VMD, Andermann E (2004). Integrative cortical dysfunction and pervasive motion perception deficit in fragile X syndrome. Neurology.

[70] Ridder WH, Borsting E, Banton T (2001). All developmental dyslexic subtypes display an elevated motion coherence threshold. Optom Vis Sci.

[71] Spencer J, O’Brien J, Riggs K, Braddick O, Atkinson A, Wattam-Bell J (2000). Motion processing in autism: evidence for a dorsal stream deficiency. Neuroreport.

[72] Vicari S, Bellucci S, Carlesimo GA (2005). Visual and spatial long-term memory: differential pattern of impairments in Williams and Down syndromes. Dev Med Child Neurol.

[73] Majerus S, Van der Linden M, Braissand V, Eliez S (2007). Verbal short-term memory in individuals with chromosome 22q11.2 deletion: specific deficit in serial order retention capacities?. Am J Ment Retard.

[74] Simon TJ, Wu ZL, Avants B, Zhang H, Gee JC, Stebbins GT. Atypical cortical connectivity and visuospatial cognitive impairments are related in children with chromosome 22q11.2 deletion syndrome. Behav Brain Funct. 2008;4(1):25. 10.1186/1744-9081-4-25 PMC2443161 18559106

[75] Radoeva PD, Coman IL, Antshel KM, Fremont W, McCarthy CS, Kotkar A, et al. Atlas-based white matter analysis in individuals with velo-cardio-facial syndrome (22q11.2 deletion syndrome) and unaffected siblings. Behav Brain Funct. 2012;8(1):38. 10.1186/1744-9081-8-38 PMC3533822 22853778

[76] Klaver P, Marcar V, Martin E (2011). Neurodevelopment of the visual system in typically developing children. Prog Brain Res.

[77] Spaniol J, Davidson PSR, Kim ASN, Han H, Moscovitch M, Grady CL (2009). Event-related fMRI studies of episodic encoding and retrieval: meta-analyses using activation likelihood estimation. Neuropsychologia.

[78] Westlye LT, Walhovd KB, Dale AM, Bjornerud A, Due-Tonnessen P, Engvig A (2010). Differentiating maturational and aging-related changes of the cerebral cortex by use of thickness and signal intensity. Neuroimage.

[79] Rentschler I, Juttner M, Osman E, Muller A, Caelli T (2004). Development of configural 3D object recognition. Behav Brain Res.

[80] Gunn A, Cory E, Atkinson J, Braddick O, Wattam-Bell J, Guzzetta A (2002). Dorsal and ventral stream sensitivity in normal development and hemiplegia. Neuroreport.

[81] Souchay C, Guillery-Girard B, Pauly-Takacs K, Wojcik DZ, Eustache F (2013). Subjective experience of episodic memory and metacognition: a neurodevelopmental approach. Front Behav Neurosci.

[82] Elliott G, Isaac CL, Muhlert N (2014). Measuring forgetting: a critical review of accelerated long-term forgetting studies. Cortex.

[83] Schneider M, Van der Linden M, Glaser B, Rizzi E, Dahoun SP, Hinard C (2012). Preliminary structure and predictive value of attenuated negative symptoms in 22q11.2 deletion syndrome. Psychiatry Res.

[84] Campbell LE, Stevens A, Daly E, Toal F, Azuma R, Karmiloff-Smith A (2009). A comparative study of cognition and brain anatomy between two neurodevelopmental disorders: 22q11.2 deletion syndrome and Williams syndrome. Neuropsychologia.

[85] Rosenthal R. Meta-analytic procedures for social research (Vol. 6). Newbury Park: Sage; 1991.

